# Supplementary figures and images for: A deep learning approach for time-consistent cell cycle phase prediction from microscopy data
Source: PLoS Comput Biol. 2025 Dec 11;21(12):e1013800. doi: 10.1371/journal.pcbi.1013800 (PMC12711074; doi:10.1371/journal.pcbi.1013800)

**A**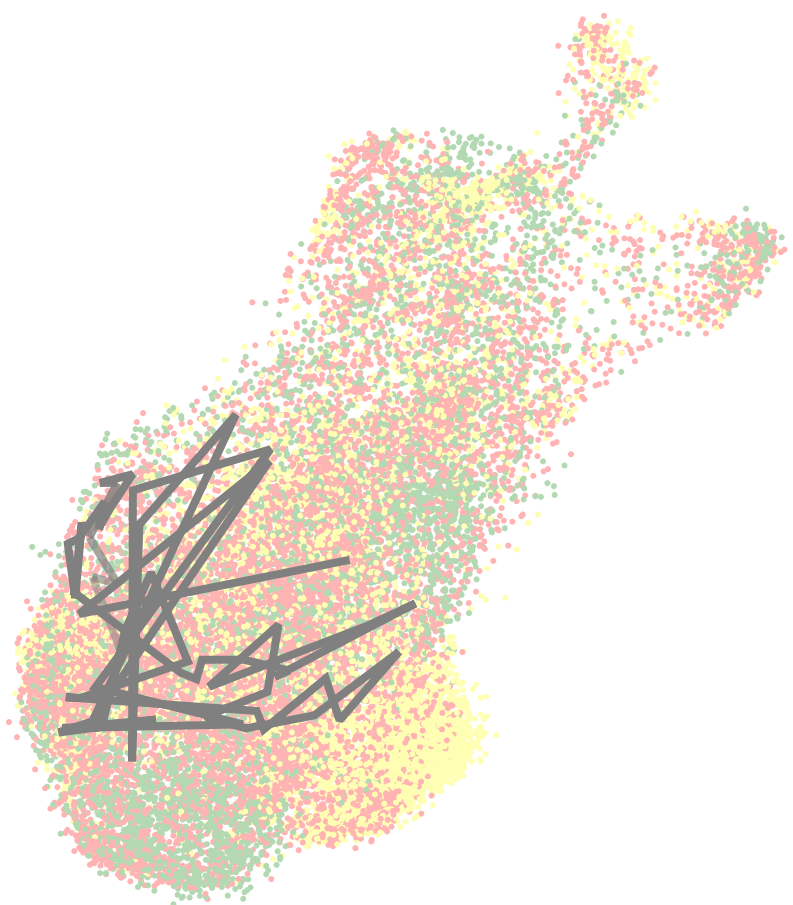**B**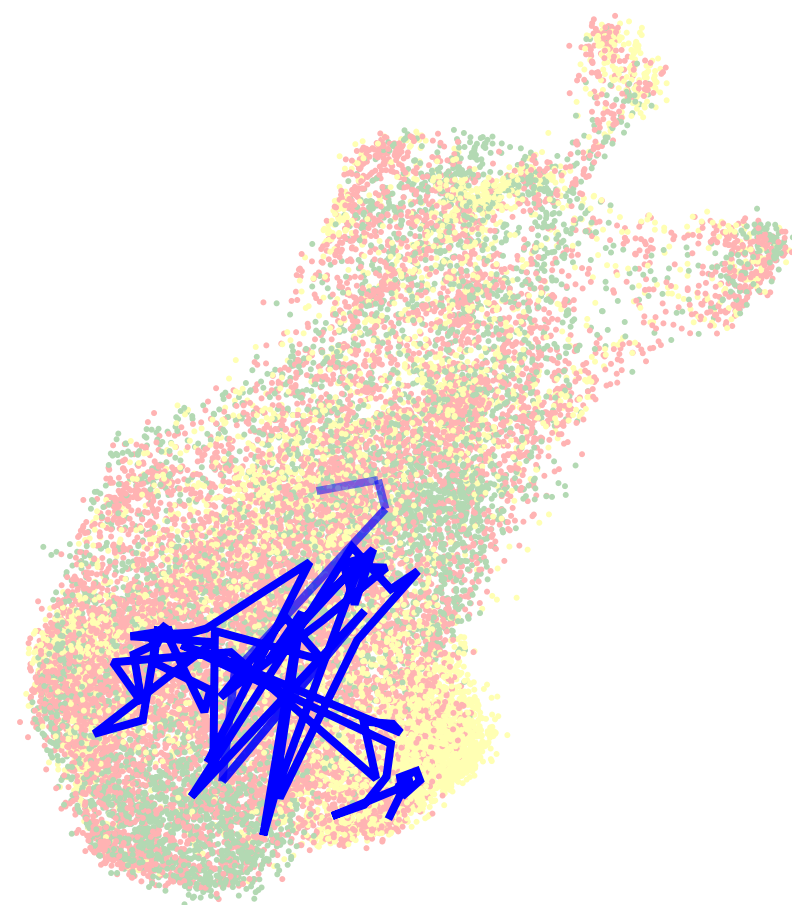**G**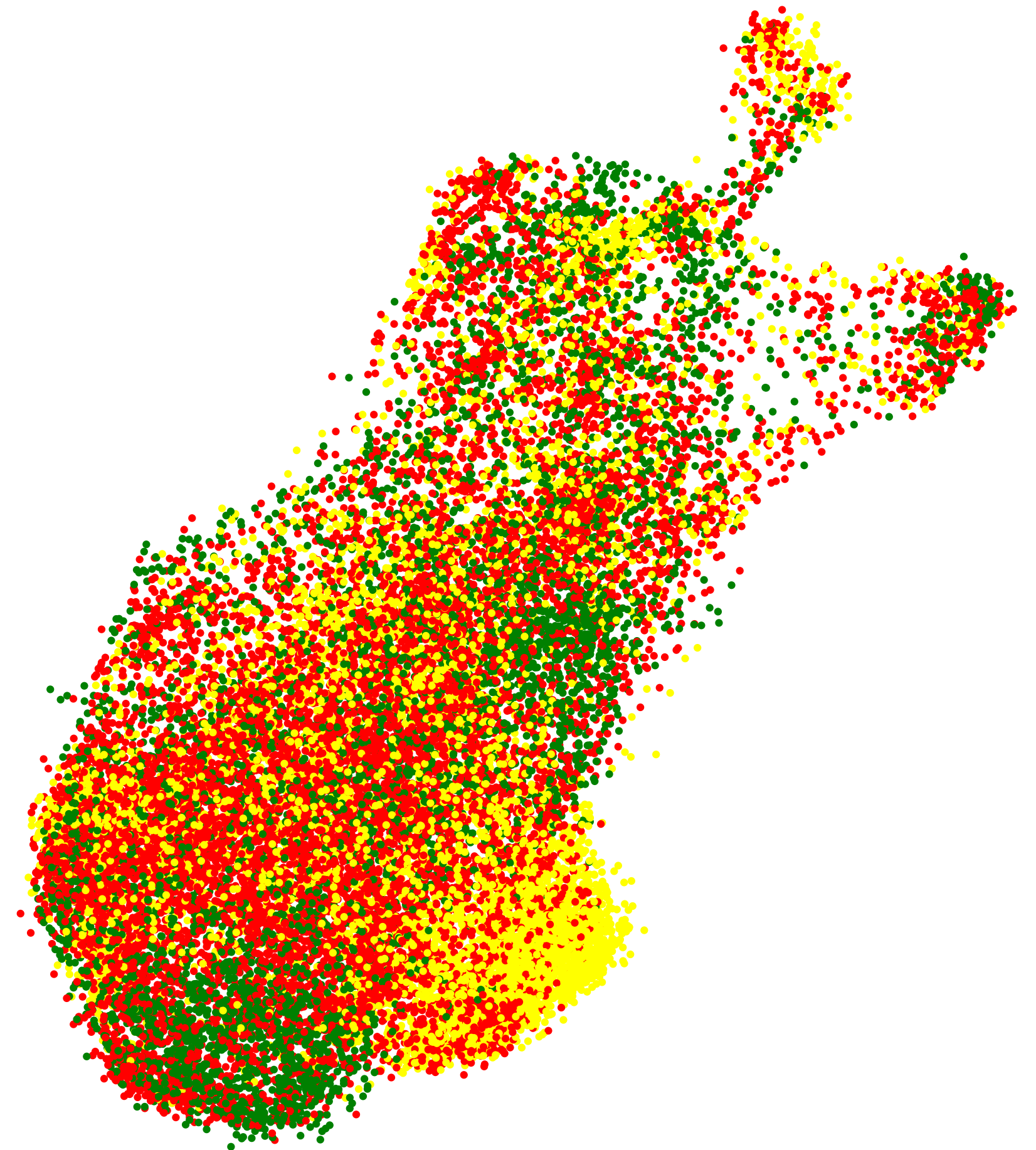**C**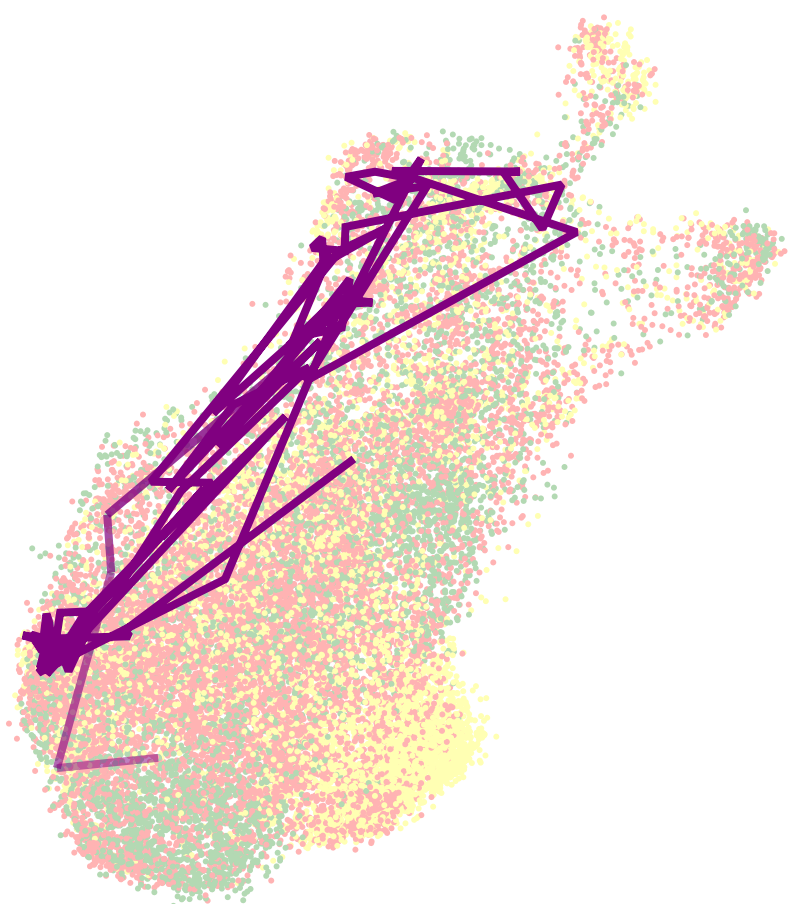**D**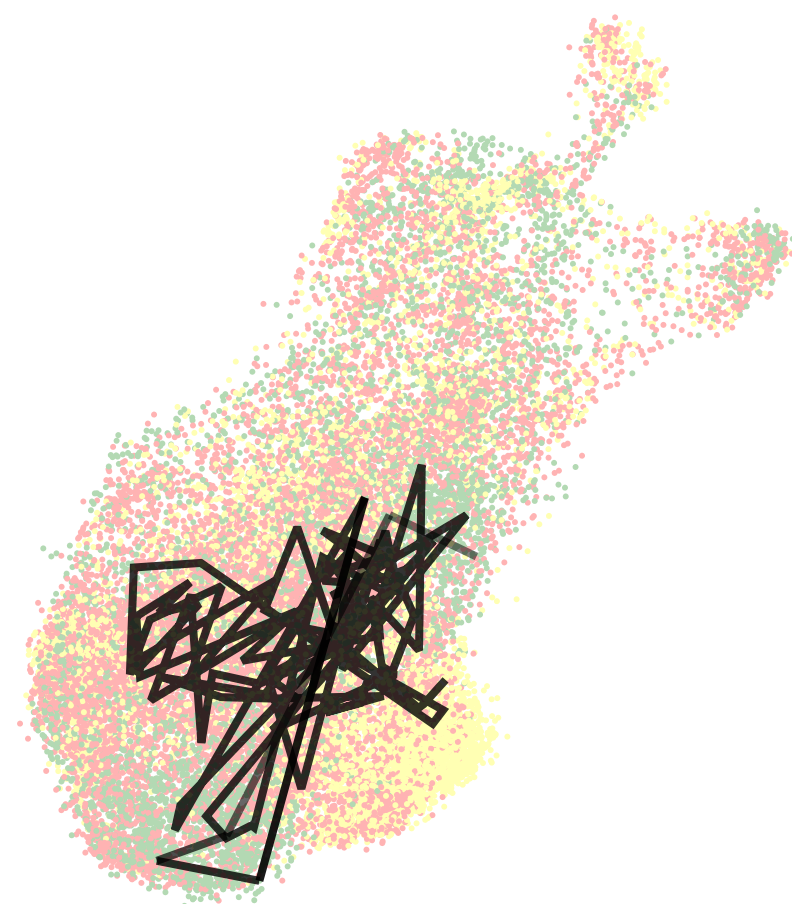**E**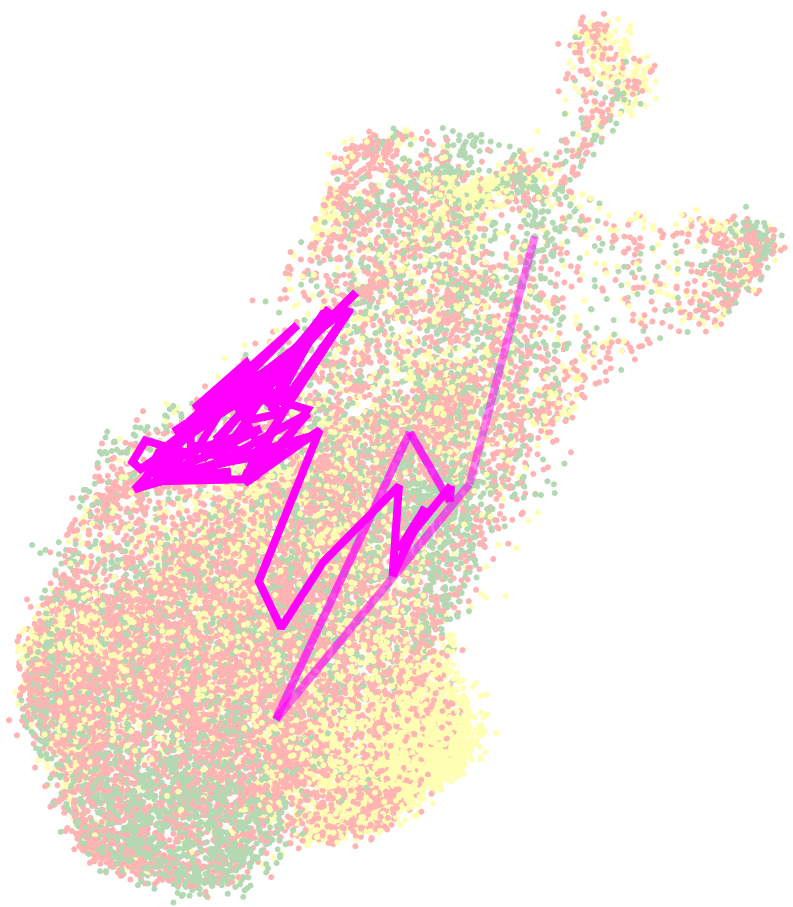**F**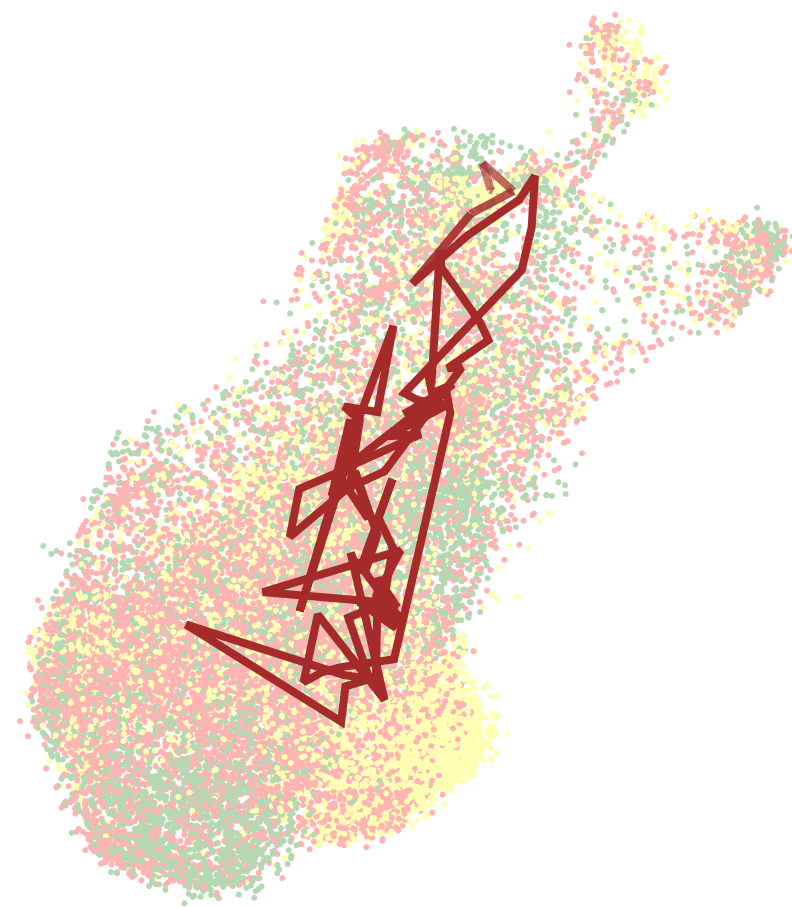**Cell cycle phase**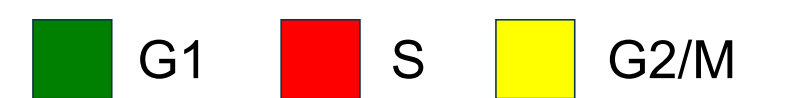

Supplement: S1 Fig — (A–F) Displayed tracks originate from the same cells as those in Fig 5. The early steps of the trajectory are rendered transparent. (G) UMAP representation of the CNN latent space. (PDF) [file pcbi.1013800.s001.pdf]

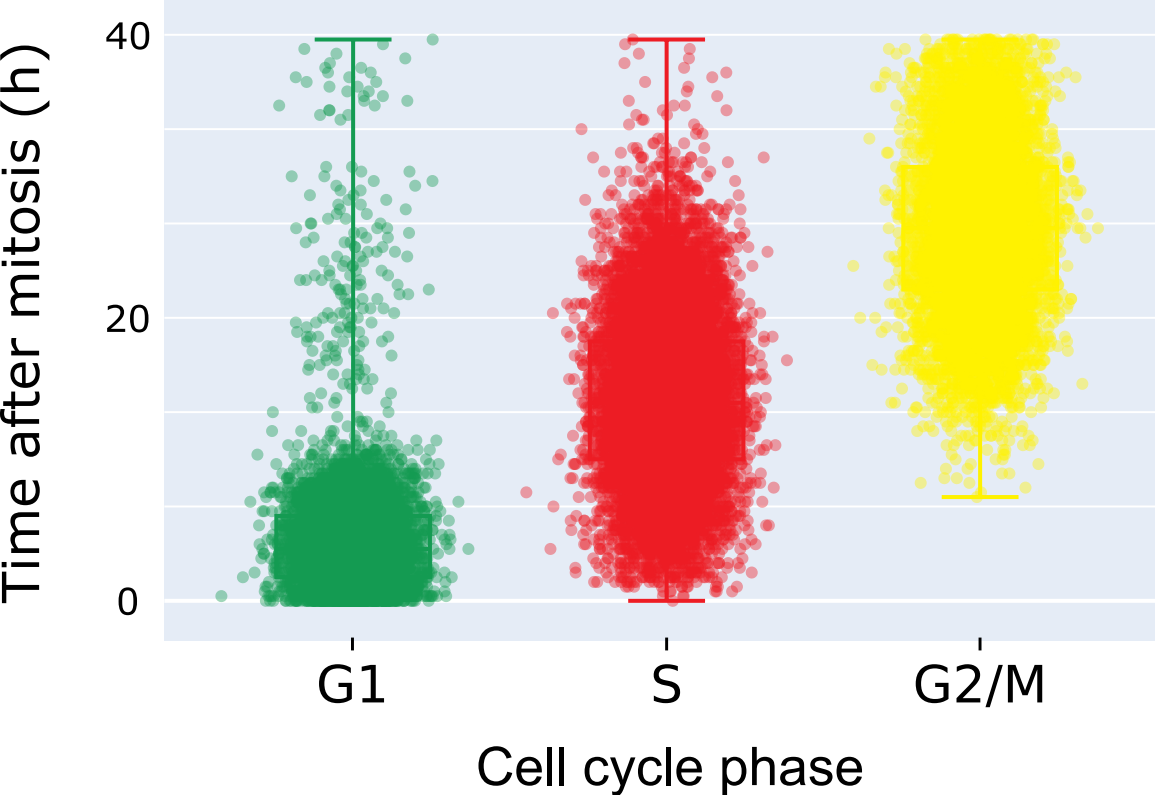

Supplement: S2 Fig — Each dot represents a nucleus from the same subset of the test set shown in Fig 3. (PDF) [file pcbi.1013800.s002.pdf]
