# Supplementary material for: A deep learning approach for time-consistent cell cycle phase prediction from microscopy data
Source: PLoS Comput Biol. 2025 Dec 11;21(12):e1013800. doi: 10.1371/journal.pcbi.1013800 (PMC12711074; doi:10.1371/journal.pcbi.1013800)

Point-by-point answer to the reviewers

We would like to thank the reviewers and the editorial team for their thoughtful and constructive feedback on our manuscript. We have carefully addressed all comments and concerns where possible, and we have conducted several additional experiments and analyses to strengthen the study. Detailed, point-by-point responses to all comments are provided below.

Reviewer 1

1. The benchmarking should also include one of the methods described in the introduction. I recommend comparing to “Ulicna et al” (reference 20) on the same dataset used for CC-VAE

We agree that comparing CC-VAE to other works presented in the introduction is an important aspect. It might not have been clear in the previous version that there we had already included some of these methods in our benchmarking:

- **SVM, as used in (Narotamo et al.) and (Huang et al.)**
- **CNN, as used in (Eulenberg et al.), (Rappez et al.), (Nagao et al.), and (Huang et al.).**

Nevertheless, we agree that comparing our approach to more recent methodologies would be valuable. In particular, we agree that the strategy proposed by *Ulcina et al.* is methodologically the most similar, and benchmarking against this method would indeed make sense. Unfortunately, *Ulcina et al.* requires time-lapse microscopy as input, whereas our method is specifically designed to predict the cell cycle phase from a single microscopy image (see detailed response below). The live-cell imaging data were only used to establish a high-confidence ground truth and to impose additional constraints during training; at inference, our method operates solely on single images.

To constructively address this concern, we evaluated our method against SimCLR (Chen et al.) and BYOL (Grill et al.), two state-of-the-art self-supervised learning strategies. SimCLR is based on contrastive learning, while BYOL employs a non-contrastive framework. In particular, BYOL relies on self-distillation, similar to the approach of (Doron et al.), also described in the introduction.

2. A proof-of-concept biological validation is missing. From the classified cell cycle phase on unseen data the authors should replicate a plot from another publication where cell cycle phase was used to obtain biological insights.

We acknowledge that such a comparison would be valuable for assessing biological validation.

We have added a plot showing the nucleus area (µm²) as a function of the predicted cell cycle phase on unseen test data. As expected, nucleus size increases progressively throughout the cell cycle, consistent with previous observations reported in the literature (Webster et al., Chambliss et al., Mukherjee et al.).

In particular, Fig 6B reproduces the trend shown in Fig 4C of Mukherjee et al. (“Simultaneously defining cell phenotypes, cell cycle, and chromatin modifications at single-cell resolution”), illustrating the increase in nuclear size from G1 to S phase and from S to G2/M.

Other comments:

3. The authors point out that the method in “Ulicna et al” requires timelapse microscopy while their method does not. However, for the contrastative learning approach, timelapse is required right? Please explain better how CC-VAE can be used with snapshot data.

The method described in (Ulicna et al.) can be separated into two stages.

In the first stage, a VQ-VAE is used to learn embeddings from single images, which are then employed in the second stage instead of the raw images. At this point, timelapse microscopy is not required.

In the second stage, Dynamic Time Warping (DTW) is applied to annotate each image as M, G1, S, or G2. DTW requires complete cell trajectories, from mitosis to mitosis. During inference, DTW aligns a reference sequence with a query sequence and uses the resulting alignment path to assign a phase label to each nucleus image in the query sequence. In short, although images are annotated individually, the annotation process depends on access to the entire sequence spanning mitosis to mitosis.

In contrast, our strategy takes the opposite approach. Training of our variational autoencoder requires timelapse microscopy, as it is built on a contrastive learning framework. However, inference operates on single-cell images, simply using a ResNet classifier pretrained with our dedicated self-supervised scheme. Hence, timelapse microscopy is used to ensure temporal consistency of the representations, and we argue that these representations are closer to the biological reality of the cell. But the single cell representations are only calculated on individual images; at inference time, they do not require live cell imaging data.

4. Along the lines of point 3, the authors seem to infer that CC-VAE has the advantage that is can be used with “standard fixed-cell microscopy data”. If true, it needs to be tested on such data.

We acknowledge that the original sentence lacked clarity. Our intention was to point out that our method operates on individual images and can thus be applied in principle to fixed-cell microscopy, provided that there is no difference in staining, conveying different image properties. We wanted to stress that we do not require time-lapse microscopy at inference time. The manuscript has been revised accordingly.

5. While I like the idea of the ablation study in Table 2, I am confused about how can the model with randomly initialized weights have such a high Top-1 retrieval accuracy?? I think this needs more careful testing, maybe multiple random initializations to get an idea of the Top-1 retrieval accuracy distribution. Otherwise, it makes me doubt the usefulness of such a metric.

The Top-1 retrieval accuracy, as defined in our study, measures how often the same cell at two consecutive time points is closer in feature space than all other cells in the batch. In practice, this reflects the temporal consistency of the learned representations.

We indeed observe that even a network with randomly initialized weights achieves a relatively high Top-1 retrieval accuracy. This can be explained by the fact that nuclei imaged at consecutive time points are visually very similar—often nearly identical—so their untrained representations remain close in feature space, even without explicit learning. In other words, even a randomly initialized CNN can produce locally consistent representations for visually similar images, not because the model encodes meaningful biological information, but because its convolutional layers respond similarly to nearly identical inputs.

What may be more surprising is that pretraining on ImageNet reduces this accuracy. Our interpretation is that ImageNet-pretrained weights constrain the embeddings to occupy a smaller, biased region of the latent space, while much of the representation capacity remains tuned for natural image features. This bias makes it harder for temporally adjacent nuclei to remain close to one another in the embedding space, leading to the observed decrease in Top-1 retrieval accuracy.

6. It is mentioned that tracking is performed with TrackMate. However, it is not clear whether the tracks were manually validated (or corrected) or not. Please clarify this. If not manually corrected, authors need to be very careful about tracking errors.

All tracks were manually verified during the cell cycle phase annotation. Although the annotation was partially automated, each of the 15,026 tracks was manually reviewed to correct potential errors. This process allowed us not only to refine phase annotations but also to identify tracking errors by detecting inconsistencies in the PIP-FUCCI signals. Overall, however, tracking quality was excellent, owing to the high temporal resolution and the strong signal-to-noise ratio provided by SiR-DNA.

7. Unfortunately, I could not test the model because I got an ImportError when running the “eval_classifier” notebook. I included the traceback error below

This issue was caused by a recent release of the segmentation_models_pytorch package. We have now forced the version in our requirements to prevent similar problems in the future. We apologize for the inconvenience.

8. The overall usability should be improved. A napari plugin would be the best, but the notebooks folder on the GitHub repo should include one notebook on how to run the classification from a single image file (possibily a TIFF file, included in the repo).

This is indeed a valuable improvement for the usability of our tool. We have added a new notebook, predict_cell_cycle_phase.ipynb, which takes a single image as input to predict its cell cycle phase. As suggested, we also included a sample image in the repository: notebooks/data/nucleus_image.tiff, which is used in the notebook.

Reviewer 2

1. It is not clear if the VAE is really needed and what exactly it brings to the table (since generation is not the aim here). What would happen if you used a vanilla autoencoder instead? I think it would valuable to add an entry to the ablation experiment table (Table 2) where $\beta=0$.

We thank the reviewer for this insightful comment. We agree that comparing CC-VAE to a simple autoencoder would be valuable to highlight the importance of the VAE.

We included a vanilla autoencoder in our ablation experiments, which differs from a VAE in that it does not involve sampling in the latent space and uses $\beta = 0$, as you noted.

As expected, the AE achieved the best reconstruction performance, since it is not burdened with the additional constraints imposed within Cell Cycle-VAE. However, it performed substantially worse in classification accuracy (0.685 ± 0.008 vs. 0.824 ± 0.004).

Here, the regularizations introduced by the VAE are not required for image generation but are essential for enforcing biological consistency within the latent space, i.e., in the representation of nuclei. Such consistency is crucial for enhancing cell cycle phase classification performance.

2. Line 96: "46,028 nuclei remained unlabeled due to the inability to reliably determine cell cycle phase boundaries". Was the model tested on these unlabeled nuclei to determine if it still works? In other words is there a significant distribution shift between the unlabeled and labeled subsets?

We tested the model on these unlabeled nuclei and included a figure (Fig 8) in the paper with illustrative examples and the corresponding UMAPs. As expected, many of the unlabeled nuclei are early-G1, which were excluded either because their low SiR-DNA signal made tracking difficult or because the absence of PIP-FUCCI transitions prevented clear phase boundary identification. However, the model appears to classify these nuclei accurately, highlighting its capability to infer the cell cycle phase on unseen data.

More generally, the unlabeled nuclei are distributed across the entire embedding space, confirming that they do not constitute a distribution distinct from the labeled nuclei.

Reviewer 3

Thank you for giving me the opportunity to review this article. The paper introduces CC-VAE, a β-Variational Auto-Encoder framework enhanced with in-silico labeling of phase-specific markers and temporal consistency regularization for cell cycle phase prediction from microscopy images stained with SiR-DNA. The authors also released a large annotated dataset (>600,000 HeLa Kyoto nuclei) along with trained models and code. The manuscript is well-written, technically sound, and reproducible, but I have several concerns that should be addressed before publication. Please find my comments below:

1. I am a bit concerned about the novelty and generalizability of this work. The integration of in-silico labeling and temporal consistency into a β-VAE is innovative, but the classification accuracy is slightly lower than that of a supervised CNN. The authors should clarify why CC-VAE is preferable when a simpler CNN performs better in accuracy, and under what conditions temporal coherence provides a practical advantage over raw performance.

We thank the reviewer for this insightful comment. Classically, the cell cycle is divided into three main phases—G1, S, and G2/M—which are the stages we aim to predict from microscopy data. However, this discretization should be interpreted with caution, as it represents a simplified view of a fundamentally continuous biological process.

Our goal is therefore not solely to maximize classification accuracy, but to learn representations that are biologically meaningful and temporally consistent. We argue that the biological plausibility of the latent space is not merely an auxiliary consideration but an intrinsic strength of the method itself, as it better reflects the continuous nature of cell cycle progression. Maintaining such biological consistency is, in our view, essential - even if it comes at the cost of a slight reduction in classification performance.

Indeed, our analysis shows that methods focusing purely on discrimination can achieve slightly higher classification accuracy (which is expected, since all network parameters are optimized for this specific objective). However, when examining the temporal trajectories of the same cells, these models exhibit erratic oscillations rather than a smooth biological progression. This indicates that their latent spaces are optimized for class separation but fail to preserve biological continuity. In contrast, CC-VAE explicitly enforces temporal coherence and yields smooth, phenotypically consistent trajectories that more accurately represent the true dynamics of the cell cycle.

2. The model is trained and evaluated solely on SiR-DNA–stained HeLa Kyoto cells from a single microscope, with no tests on other stains, cell types, or platforms. Including external datasets, cross-cell-line validation, or transfer-learning experiments would address generalization concerns and strengthen the claims of broader applicability.

We agree that evaluating CC-VAE on other data sets from different experimental settings would be valuable. The reason we did not include additional experiments using other microscopes or platforms is that, to the best of our knowledge, no other public dataset contains HeLa cells labeled with SiR-DNA fluorescence.

That said, transfer learning experiments would indeed be valuable, as they could allow our model to generalize to other cell types or fluorescent stains. However, we consider this an extension beyond the scope of the present work, which is focused on introducing our method alongside the publication of the dataset on which it was trained. Nevertheless, we view this as a promising direction for future research.

3. The latent space visualizations are compelling, but misclassifications (particularly at G1/S and S/G2/M transitions) are not well explained in biological terms. An analysis linking errors to chromatin or morphological features would increase trust in the model among biological users.

We thank the reviewer for this comment. In answer to this suggestion, we have added a new figure (Fig 7) dedicated to linking classification errors to morphological features at the G1/S and S/G2/M transitions. While CC-VAE achieves high accuracy in cell cycle phase classification, it is not entirely free from errors, particularly at phase boundaries.

We analyzed these misclassifications with respect to nuclear area, a feature commonly used to assess cell cycle phase (Webster et al., Mukherjee et al.). Most errors involve large S-phase nuclei misclassified as G2/M, and small early S-phase nuclei incorrectly labeled as G1. Because their nuclear areas slightly deviate from the typical phase distributions, CC-VAE had difficulty classifying them with complete confidence.

4. The evaluation is limited to a morphology-based SVM and a supervised CNN. The authors should benchmark against recent self-supervised methods such as SimCLR, BYOL, MoCo, DINO, or masked autoencoders to better assess whether CC-VAE offers advantages over current deep learning approaches.

We thank the reviewer for this comment. In answer to this suggestion, we have included additional experiments in our benchmark, comparing CC-VAE with SimCLR (Chen et al.) and BYOL (Grill et al.). Both SimCLR and BYOL represent state-of-the-art self-supervised learning strategies. SimCLR is based on contrastive learning, while BYOL employs a non-contrastive framework. In particular, BYOL relies on self-distillation, similar to the approach of (Doron et al.), also described in the introduction. The results presented in Table 1 indicate that CC-VAE compares favorably to both methods.

5. The introduction should provide a stronger motivation for the study by clearly stating the research gap it addresses.

While the cell cycle phase is linked to many cellular processes (gene expression, transcriptional activity, drug sensitivity, among others), biologists currently lack a robust method for determining them without relatively complex setups or dedicated markers. We argue that AI empowered cell cycle stratification can enrich phenotypic readouts, and allow correlating phenotypic observations to cell cycle progression. In this study, we address this gap by proposing a representation learning approach to determine the cell cycle phase from a DNA marker. This motivation has been further developed throughout the paper.

Furthermore, and compared to previous work, we propose a regularized latent space that enforces biological consistency. The method therefore also provides better representations for cell nuclei. We specifically address this aspect also in the introduction to better motivate our methodological contribution.

6. The objectives of the research should be explicitly outlined at the end of the introduction.

At the end of the introduction, we have added that “our goal is to provide the community with a robust, simple, and open-source method for cell cycle phase classification, along with a large publicly available nucleus image dataset to support further research”. Furthermore, as explained in answer to concern (5), we also explain more clearly that we also aim at representation learning, ensuring not only classification encuracy, but also biological consistency of the latent space.

7. At the end of the introduction, add the organization of the paper.

The organization of the paper has been added at the end of the introduction.

8. Format the paper correctly. The paper does not follow the standard PLOS Computational Biology structure. The Methods section comes after the Results and Discussion, making it hard for readers to understand the study before seeing the findings. Reorder sections as: Introduction → Methods → Results → Discussion → Conclusion.

We have followed the standard PLOS Computational Biology structure, as outlined here: https://journals.plos.org/ploscompbiol/s/submission-guidelines#loc-manuscript-organization. According to these guidelines, the sections should be organized as follows: Introduction → Results → Discussion → Methods. However, we included the presentation of our model, "Overview of CC-VAE," within the Results section to introduce the key concepts underlying our methodology before presenting the actual results.

9. The methodology section misses some important points.
a) How exactly are the temporal contrastive pairs selected in cases of irregular cell cycle timing?

Training is performed on sequences of nucleus images. For each image, positive pairs are generated by associating it with the images of the same nucleus at the immediately preceding and following time points. If one of these neighboring frames is unavailable (e.g., for the first or last frame of a sequence), only a single positive pair is used. Negative pairs are formed by pairing the reference image with any other nucleus image within the same batch. We have clarified these details in the revised manuscript.

Importantly, the construction of contrastive pairs does not depend on cell cycle phase labels. In other words, we do not exclude pairs corresponding to nuclei at phase-transition points. As a result, variations in cell cycle timing have no effect on the contrastive learning procedure—the model simply encourages temporally adjacent images of the same nucleus to occupy nearby positions in the latent space, regardless of where they fall within the cell cycle.

b) Whether all z-stack planes were used or maximum projections were applied before training.

All z-stack planes were used. This information has been added to the manuscript.

c) Normalization applied to the SiR-DNA intensity before input to the network.

SiR-DNA images were indeed normalized before being provided as input to the model. This information has been added to the manuscript.

10. Several figures could be improved for clarity:
a) In Fig. 4, arrows and cluster labeling in the UMAP could be made clearer for readers unfamiliar with embedding spaces.

To improve cluster readability for readers unfamiliar with embedding spaces, we highlighted the areas of interest using the same color as the arrows. This allows the referenced nuclei to be identified more clearly.

b) In Fig. 5, adding a temporal color gradient would help visualize progression along the cell cycle trajectory.

We thank the reviewer for this suggestion. Indeed, a temporal gradient helps visualize progression along the cycle. We have modified the figure by changing transparency of the trajectory such that early steps of the trajectory are transparent, while the later steps remain opaque.

11. While an ablation study is included, a more explicit sensitivity analysis (e.g., effect of β parameter, weight of temporal loss, or latent dimensionality) would improve transparency about the robustness of design choices.

Indeed, such an analysis would be valuable for gaining a deeper understanding of the hyperparameter selection process. In the Methods section, we have included full details and results from our hyperparameter search. This includes classification results obtained for different values of β, the self-supervised learning rate, latent dimensionality, and λ_1 and λ_2 (weight of the temporal loss).

Notably, we observe that excessively high values of β negatively affect classification accuracy, consistent with previous findings (Chadebec et al.). Furthermore, larger latent spaces generally improve classification performance, with improvements plateauing around dimensions of 256–512.

Reviewer 4

The authors present the Cell Cycle Variational Auto-Encoder (CC-VAE), a novel self-supervised deep learning approach designed to determine the cell cycle phase of individual cells based on a DNA fluorescence marker. According to the manuscript, CC-VAE provides a powerful tool for stratifying cells by their cycle phase, thereby facilitating the investigation of potential links between the cell cycle and patterns of RNA or protein localization.
Temporal consistency is particularly important because the cell cycle is a continuous biological process, not a set of isolated events. Although for analytical convenience the cycle is often discretized into three main phases (G1, S, G2/M), in reality the transitions between them occur gradually, with overlapping molecular and morphological signatures. Ensuring temporal consistency in modelling therefore better reflects the underlying biology, prevents artificial discontinuities introduced by phase labeling, and improves the accuracy of downstream analyses such as tracking RNA or protein localization dynamics.
Overall, the authors have done a commendable job in preparing this manuscript - the research is solid, the results are clearly presented, and the language is well written.

We sincerely thank the reviewer for the positive and encouraging assessment of our work. We fully agree that the traditional discretization of the cell cycle into distinct phases represents an analytical simplification of a fundamentally continuous biological process. Our approach was specifically designed with this in mind, as it aims to preserve temporal and phenotypic continuity rather than relying solely on discrete phase boundaries.

However, there are some minor issues that I would like them to address.

Fig. 1 shows that the G1 phase is represented by SiR-DNA and PIP-mVenus.
In the main text, the authors state: “Fig. 1 displays some example nuclei from our annotated dataset. G1 nuclei express only PIP-mVenus fluorescence, S nuclei express only mCherry-Gem1-110 fluorescence, and G2 nuclei express both signals” (lines 100–102).
Was there any difference in the testing of cells that expressed only SiR-DNA, only PIP-mVenus, or both together, if such alone-expression is possible?

We thank the reviewer for raising this point and for the opportunity to clarify. SiR-DNA is a DNA marker present in all nuclei, regardless of the cell cycle phase, as shown in Fig. 1. PIP-mVenus, together with mCherry-Gem1-110, forms the PIP-FUCCI system, which is commonly used to identify cell cycle phases. Specifically, as stated in the manuscript: *“G1 nuclei express only PIP-mVenus fluorescence, S nuclei express only mCherry-Gem1-110 fluorescence, and G2 nuclei express both signals.”* By “only,” we mean, for example, that G1 nuclei express PIP-mVenus but not mCherry-Gem1-110.

PIP-mVenus and mCherry-Gem1-110 together provide the ground-truth labels for the cell cycle phases, which we curate from time-lapse microscopy data. However, at inference time, our classifier uses only SiR-DNA fluorescence images as input to predict the cell cycle phase—without relying on any PIP-FUCCI markers.

Line 407: please describe, why 10 epochs was used to train the model? Was it based on the performance (loss check)?

We thank the reviewer for raising this point. We chose to train our model for 10 epochs based on performance. Below, we display the loss evolution across the 10 cross-validation folds of our training, monitored using Weights & Biases. As observed, the loss decreases during the initial epochs and then tends to plateau, consistently across all runs. The choice of 10 epochs

was made to allow sufficient time for this plateau to be reached, combined with early stopping to select the best-performing model for inference on the test data.

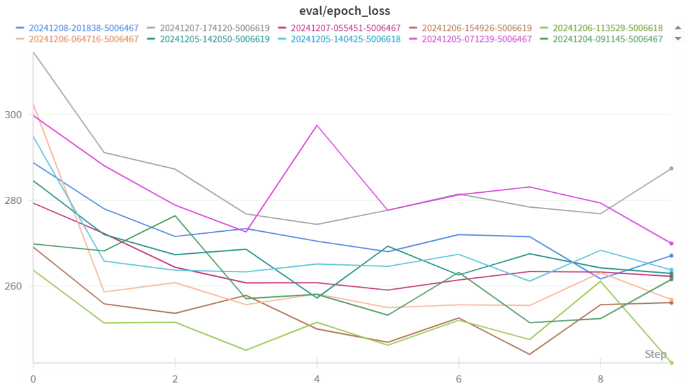

Line 420: Similar as above. The authors report that supervised classification training was performed for only one epoch with a learning rate of 1e-4 and batch size of 64. While early stopping was employed, it is unclear whether training for a single epoch is sufficient for the network to converge, particularly given that the classifier contains ~67k parameters. Typically, training deep models for such tasks requires multiple epochs to allow the optimization process to properly minimize the loss function and avoid underfitting. It would be important to clarify whether (i) additional epochs were tested, (ii) the training/validation curves indicated convergence within a single epoch, and (iii) performance stabilized consistently across runs. Otherwise, there is a risk that the reported accuracy reflects insufficient training rather than the true capacity of the model.

We acknowledge that training deep learning models for only a single epoch is unusual. However, in our case, we trained only the 67k parameters of the classifier on a dataset of more than 500k nucleus images, which led to a very rapid convergence of the loss curves.

Below, we show the training cross-entropy loss evolution across the 10 cross-validation folds, monitored with TensorBoard. The x-axis represents the number of steps, where each step corresponds to one batch of 64 images. The loss decreases sharply during the initial steps and quickly reaches a plateau. Since this plateau was consistently reached within the first epoch, we stopped training at that point.

This curve addresses your comments: (i) we did not test additional epochs, as convergence was achieved well before the end of the first epoch; (ii) the training curve clearly shows convergence within this single epoch; and (iii) performance was consistent across all 10 runs.

train/CrossEntropyLoss
tag: train/CrossEntropyLoss

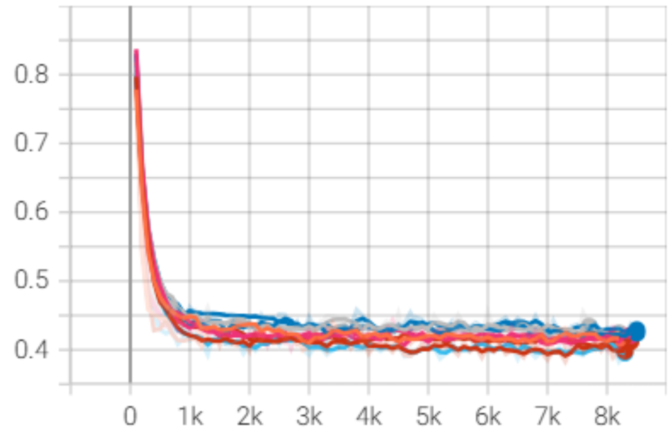

Supplement: Attachment — Submitted filename: Review CC-VAE.pdf [file pcbi.1013800.s004.pdf]
