# Supplementary material for: A deep learning approach for time-consistent cell cycle phase prediction from microscopy data
Source: PLoS Comput Biol. 2025 Dec 11;21(12):e1013800. doi: 10.1371/journal.pcbi.1013800 (PMC12711074; doi:10.1371/journal.pcbi.1013800)
Supplement: Attachment — Submitted filename: Review_PCOMPBIOL-D-25-01257.docx [file pcbi.1013800.s003.docx]

**Manuscript Number: PCOMPBIOL-D-25-01257**

**Title: A Deep Learning approach for time-consistent cell cycle phase prediction from microscopy data**

Thank you for giving me the opportunity to review this article. The paper introduces CC-VAE, a β-Variational Auto-Encoder framework enhanced with in-silico labeling of phase-specific markers and temporal consistency regularization for cell cycle phase prediction from microscopy images stained with SiR-DNA. The authors also released a large annotated dataset (>600,000 HeLa Kyoto nuclei) along with trained models and code. The manuscript is well-written, technically sound, and reproducible, but I have several concerns that should be addressed before publication. Please find my comments below:

**1.** I am a bit concerned about the novelty and generalizability of this work. The integration of in-silico labeling and temporal consistency into a β-VAE is innovative, but the classification accuracy is slightly lower than that of a supervised CNN. The authors should clarify why CC-VAE is preferable when a simpler CNN performs better in accuracy, and under what conditions temporal coherence provides a practical advantage over raw performance.

**2.** The model is trained and evaluated solely on SiR-DNA–stained HeLa Kyoto cells from a single microscope, with no tests on other stains, cell types, or platforms. Including external datasets, cross-cell-line validation, or transfer-learning experiments would address generalization concerns and strengthen the claims of broader applicability.

**3.** The latent space visualizations are compelling, but misclassifications (particularly at G1/S and S/G2/M transitions) are not well explained in biological terms. An analysis linking errors to chromatin or morphological features would increase trust in the model among biological users.

**4.** The evaluation is limited to a morphology-based SVM and a supervised CNN. The authors should benchmark against recent self-supervised methods such as SimCLR, BYOL, MoCo, DINO, or masked autoencoders to better assess whether CC-VAE offers advantages over current deep learning approaches.

**5.** The introduction should provide a stronger motivation for the study by clearly stating the research gap it addresses.

**6.** The objectives of the research should be explicitly outlined at the end of the introduction.

**7.** At the end of the introduction, add the organization of the paper.

**8.** Format the paper correctly. The paper does not follow the standard PLOS Computational Biology structure. The Methods section comes after the Results and Discussion, making it hard for readers to understand the study before seeing the findings. Reorder sections as: Introduction → Methods → Results → Discussion → Conclusion.

**9.** The methodology section misses some important points.

a) How exactly are the temporal contrastive pairs selected in cases of irregular cell cycle timing?
b) Whether all z-stack planes were used or maximum projections were applied before training.
c) Normalization applied to the SiR-DNA intensity before input to the network.

**10.** Several figures could be improved for clarity:

a) In Fig. 4, arrows and cluster labeling in the UMAP could be made clearer for readers unfamiliar with embedding spaces.
b) In Fig. 5, adding a temporal color gradient would help visualize progression along the cell cycle trajectory.

**11.** While an ablation study is included, a more explicit sensitivity analysis (e.g., effect of β parameter, weight of temporal loss, or latent dimensionality) would improve transparency about the robustness of design choices.
