# Supplementary material for: A deep learning approach for time-consistent cell cycle phase prediction from microscopy data
Source: PLoS Comput Biol. 2025 Dec 11;21(12):e1013800. doi: 10.1371/journal.pcbi.1013800 (PMC12711074; doi:10.1371/journal.pcbi.1013800)
Supplement: Attachment — Submitted filename: Review_PCOMPBIOL-D-25-01257.docx [file pcbi.1013800.s005.docx]

**Manuscript Number: PCOMPBIOL-D-25-01257**

**Title: A Deep Learning approach for time-consistent cell cycle phase prediction from microscopy data**

All my comments have been fully addressed in the revised manuscript. The authors added new experiments, clarified methodological details, expanded the introduction, improved usability, and updated figure quality. I recommend acceptance.
